# Peri-operative data on the nuss procedure in children with pectus excavatum: independent survey of the first 20 years' data

**DOI:** 10.1186/1749-8090-3-40

**Published:** 2008-07-04

**Authors:** Aristotle D Protopapas, Thanos Athanasiou

**Affiliations:** 1 Department of Biosurgery & Surgical Technology, Imperial College London, St. Mary's Hospital, London, W2 1NY, UK

## Abstract

**Objective:**

To review the literature and assess the cumulative data on the Nuss operation in children on its twenty years' anniversary: The Nuss procedure corrects the pectus excavatum by minimal access semi-permanent insertion of metal bars in order to reduce the deformity and refashion the contour of the growing thorax. The advantage over previous techniques is avoidance of osteochondrotomies and thence allowance for normal growth of the thorax.

**Study design:**

PubMed search was performed. Primary outcomes were mortality, morbidity and individual complications. Secondary outcomes were procedure time and hospital stay.

**Results:**

We merged the data from 19 reports comprising 1949 children of mean age 10.6 years.

No mortality was observed and the procedure was associated with morbidity of 15.4%. The commonest complications are bar-related adverse events (5.7%) and pneumothorax (3.5%). The average procedure time and the average hospital stay were 68 minutes and 5.5 days respectively.

**Conclusion:**

20 years of initial evidence suggests that the Nuss group of procedures is a safe minimal access option for correction of pectus excavatum in childhood.

## Introduction

The cardiothoracic surgeons are moving towards minimally invasive techniques. Such a technique is the Nuss repair (alias Minimally Invasive Repair of Pectus Excavatum or Miniature Access Pectus Excavatum Repair) for pectus excavatum (funnel chest) [1], the commonest chest wall anomaly in humans [2], first described in 1594 by Johannes Schenk, occurring in approximately 1 in every 400 births, males being afflicted 5 times more often than females. The indication for correction is primarily cosmetic, although the potential for cardiorespiratory improvement can be considered.

The original Nuss technique has being previously described [1,24]. Its principle is the permanent reduction of the bone deformity by insertion of one (or more) malleable metal bars in order to refashion the contour of the growing thorax.

Advantages and disadvantages of the Nuss in relation to open techniques (such as Ravitch [2] and Willital-Hegemann that include extensive thoracic incisions and multiple thoracic osteochondrectomies (resections of ribs and cartilage) are presented in Table 1.

**Table 1:** Perceived advantages and disadvantages of minimal access strategy for correction of pectus in childhood in comparison to pre-existing conventional techniques

Advantages	Disadvantages
Short hospital stay	Cost of thoracoscopy and equipment
Minimal trauma	Second procedure for bar removal
Allowance for skeletal growth	Capnothorax in thoracoscopy

The principal advantage over these techniques is avoidance of osteochondrotomies and thence allowance for normal growth of the thorax, as subperichondral resection of the costal cartilages may halt the growth of the thoracic cage in toddlers and adolescents.

The metalwork is later removed as a day-case operation (nor requiring overnight stay in hospital) under general anaesthesia.

The Nuss operation can be performed with or without use of thoracoscopy. The selection of age for the Nuss varies with clinical, personal and socio-economical reasons (such as change of school and fear of intimidation by new peers), while removal of bars is scheduled within two to three years from the insertion. In Britain, some surgeons prefer to perform Nuss around the age of 10, before the child changes schools and thence is exposed to new peers. Some other surgeons will perform Nuss earlier, deciding on parental preference and individual clinical circumstances.

## Materials and methods

We searched the literature with a simple strategy : 

PubMed search   

Last Date performed: 31 December 2006

Search keyword ‘Nuss’, language English, Humans, children  

Cross-validation by hand search to identify case series and exclude isolated case reports.   

Primary outcomes: Mortality, morbidity, individual complications

Secondary outcomes: Procedure time and hospital stay.  

Descriptive and summary statistics were performed. Denominators were related to actual data. Missing data were not defaulted.

## Results

### Selection of reports

18 series of Nuss on children were identified (Table 2), originating from one or more of seven countries, or one of five of the United States of America.

**Table 2:** The series merged, 20 years of Nuss operations in children 1987–2006

**Reference** **number**	**Patients** **operated**	**Type of study**	**Number of ** **centres**	**Comment**
1	329	Retrospective	One	Series update on ref. 24
3	21	Comparative	One	
4	52	Retrospective	One	
5	335	Retrospective	One	Encompasses ref. 19
6	53	Retrospective	One	
10	22	Retrospective	One	
11	40	Retrospective	Not reported	
12	172	Retrospective	Eight	
13	31	Retrospective	One	
15	20	Retrospective	One	Modified technique
16	36	Comparative	One	
23	27	Retrospective	One	Subgroup of all-age cohort
8*	107	Comparative	One	Similar data to ref. 9
9*	107	Retrospective	Not reported	Similar data to ref. 8
14	80	Comparative	One	
17**	35	Comparative	One	Same centre as ref. 18
18**	21	Retrospective	One	Same centre as ref. 17
22	461	Retrospective	One	

**Total**	**1949**			

Of these, there were at least three reports preceded by others with apparently overlapping cohorts, [2] by [20,3] by [13] and [14,5] by [19] so we utilised data from the larger and more up to date ones [2,3,5].

Interestingly, two reports from neighbouring countries [Japan, South Korea, [8,9]) over a similar period had the same number of subjects (107 each), similar but not identical demographics (age, gender) and similar outcomes. Both reports have being included separately in our survey. Two reports from the same centre seemed to report on separate cohorts [17,18] and have being also included separately in our survey.

### Demographics (Table 3)

**Table 3:** Cumulative perioperative data on 20 years of Nuss operations in children 1987–2006

**Reference** ** number**	**Patient** **number**	**Average Age**	**Average** **Operating** ** Time**	**Average** **Hospital Stay**
1	329	11 years	Not reported	5 days
3	21	14.4 years	53'	Not reported
4	52	Unknown	106'	3.9 days
5	335	8 years	Not reported	Not reported
6	53	9 years	76'	8.9 days
10	22	15.5 years	Not reported	13.4 days
11	40	17.6 years	126'	Not reported
12	172	15.1 years	76'	Not reported
13	31	14.5 years	Not reported	4 days
15	20	14 years	75'	5.5 days
16	36	12.3 years	96'	5.5 days
8	107	7.9 years	67'	8 days
9	107	7.5 years	48'	Not reported
14	80	11.5 years	53'	3.7 days
17	35	9.5 years	198'	4.8 days
18	21	8.2 years	Not reported	4.9 days
22	461	15.2 years	52'	5.3 days
23	27	5.9 years	52'	4.9 days

**Total**	**1949**	**10.6 years**	**68'**	**5.5 days**

1949 children have had Nuss operations. Mean age was 10.6 years, ratio male: female 77:23.

### Morbidity and Mortality

No mortality was observed and the incidence of morbidity was 15.4%. The most commonly reported complications were:

1. *Bar-related events (bar displacement requiring revision) *(111 events, incidence 5.7%) and

2. *Pneumothorax *(68 events including those treated without chest drain, overall incidence 3.5%).

The incidence of wound infection was 2.2%, the incidence of other pleuropulmonary complications including effusions and atelectasis/pneumonia was 2%. Other complications were less common (Table 4).

**Table 4:** Complications of 20 years of Nuss operations in children 1987–2006

**Complication**	**Cumulative**
Bar-related adverse events	111 (37%)
Pneumothorax	68(23%)
Other Pleuropulmonary, except pneumothorax	39(13%)
Wound infection	43(14%)
Pericardial effusion	28(9%)
Hemothorax	12(4%)

**Total**	**301**

### Other Perioperative Data

The average length of operation in minutes was 68 minutes (range 28–200).

Average Hospital stay was 5.5 days (range 2–27 days).

## Conclusion

We hope that this brief independent survey will offer the necessary peri-operative data on this now well-established cosmetic intervention in children: The Nuss procedure has been performed all around the world with no reported mortality for 20 years (1987–2007), indicated primarily for cosmesis in the paediatric sufferer of pectus excavatum. Potential cardiorespiratory improvement is not as yet confirmed, whilst the co-existence of Marfan's syndrome can be ruled out by pre-operative echocardiography.

The variations of the Nuss procedure stem from thoracoscopic or open, and then thoracoscopy with single or double-lumen ventilation (in toddlers double lumen ventilation may be cumbersome given their tracheal size). Bar stabilisers have evolved as a valid addition to the technique [11].

Pneumothorax and bar-related events (pain, dislocation or infection) may complicate the procedure and are the primary post operative points of concern. Pneumothorax is as expected, commoner with thoracoscopy: the technique may involve carbon dioxide insufflation (capnothorax [25] where single lumen tracheal intubation is utilised.

Our observations reinforce these of a previous smaller multi-centre cumulative report on 251 cases 7 years ago [21] and a recent case review by the inventor of the technique [24].

The advantages of this procedure include the following: the short hospital stay and limited invasion surgery which allows for growth in the skeleton as opposed to the ostochondrectomies (Table 1). On the balance is the obvious cost of the thoracoscopy and specialised equipment as well as the second outpatient-day case procedure of removal of the bar(s).

We have now reached the point of adequate experience with Nuss that the purchasers may decide on strategies after careful individual cost-effectiveness assessment. Most workers timed the operation at an age appropriate to the cosmetic expectations of the patient and family considering the growth spurt of teenagers, namely prior to the early teens. It is not unusual to perform Nuss in young adults as a matter of surgeon's and patient's preference, where care should be exercised for the bar recipient not to be exposed to vigorous activity prior to removal of the bar as displacement is a recognised complication associated with contact sports, trauma or intense manual labour[26].

## Limitations of the study and future research

Not all reported series include the data for the variables studied, the length of postoperative in-hospital stay being one important one. This might have an impact on the results. Post operative hospital stay is a surrogate index of performance, especially in paediatric populations. It is evident in the literature that the available data have not been based in comparative high quality studies and patient based outcomes such as Health Related Quality of life and patient satisfaction which are important considerations in therapeutic decision making. Also the long-term results of the procedure are not being discussed in this paper.

## Competing interests

The authors declare that they have no competing interests.

## Authors' contributions

AP conceived the research idea and drafted the manuscript. TA corrected the manuscript. Both authors read and approved the final manuscript.

## References

[B1] Nuss D, Croitoru DP, Kelly RE, Goretsky MJ, Nuss KJ, Gustin TS (2002). Review and discussion of the complications of minimally invasive pectus excavatum repair. Eur J Pediatr Surg.

[B2] Huddleston CB (2004). Pectus excavatum. Semin Thorac Cardiovasc Surg.

[B3] Boehm RA, Muensterer OJ, Till H (2004). Comparing minimally invasive funnel chest repair versus the conventional technique: an outcome analysis in children. Plast Reconstr Surg.

[B4] Zallen GS, Glick PL (2004). Miniature access pectus excavatum repair: Lessons we have learned. J Pediatr Surg.

[B5] Park HJ, Lee SY, Lee CS (2004). Complications associated with the Nuss procedure: analysis of risk factors and suggested measures for prevention of complications. J Pediatr Surg.

[B6] Watanabe A, Watanabe T, Obama T, Ohsawa H, Mawatari T, Ichimiya Y (2004). The use of a lateral stabilizer increases the incidence of wound trouble following the Nuss procedure. Ann Thorac Surg.

[B7] Ohno K, Morotomi Y, Ueda M, Yamada H, Shiokawa C, Nakaoka T (2003). Comparison of the Nuss procedure for pectus excavatum by age and uncommon complications. Osaka City Med J.

[B8] Jo WM, Choi YH, Sohn YS, Kim HJ, Hwang JJ, Cho SJ (2003). Surgical treatment for pectus excavatum. J Korean Med Sci.

[B9] Uemura S, Nakagawa Y, Yoshida A, Choda Y (2003). Experience in 100 cases with the Nuss procedure using a technique for stabilization of the pectus bar. Pediatr Surg Int.

[B10] Haecker FM, Bielek J, von Schweinitz D (2003). Minimally invasive repair of pectus excavatum (MIRPE) – the Basel experience. Swiss Surg.

[B11] Schaarschmidt K, Kolberg-Schwerdt A, Dimitrov G, Straubeta J (2002). Submuscular bar, multiple pericostal bar fixation, bilateral thoracoscopy: A modified Nuss repair in adolescents. J Pediatr Surg.

[B12] Hosie S, Sitkiewicz T, Petersen C, Gobel P, Schaarschmidt K, Till H (2002). Minimally invasive repair of pectus excavatum – the Nuss procedure. A European multicentre experience. Eur J Pediatr Surg.

[B13] Jacobs JP, Quintessenza JA, Morell VO, Botero LM, van Gelder HM, Tchervenkov CI (2002). Minimally invasive endoscopic repair of pectus excavatum. Eur J Cardiothorac Surg.

[B14] Miller KA, Woods RK, Sharp RJ, Gittes GK, Wade K, Ashcraft KW (2001). Minimally invasive repair of pectus excavatum: a single institution's experience. Surgery.

[B15] Hebra A, Gauderer MW, Tagge EP, Adamson WT, Othersen HB (2001). A simple technique for preventing bar displacement with the Nuss repair of pectus excavatum. J Pediatr Surg.

[B16] Wu PC, Knauer EM, McGowan GE, Hight DW (2001). Repair of Pectus Excavatum Deformities in Children: A New Perspective of Treatment Using Minimal Access Surgical Technique. Arch Surg.

[B17] Molik KA, Engum SA, Rescorla FJ, West KW, Scherer LR, Grosfeld JL (2001). Pectus excavatum repair: experience with standard and minimal invasive techniques. J Pediatr Surg.

[B18] Engum S, Rescorla F, West K, Rouse T, Scherer LR, Grosfeld J (2000). Is the grass greener? Early results of the Nuss procedure. J Pediatr Surg.

[B19] Park HJ, Lee SY, Lee CS, Youm W, Lee KR (2004). The Nuss procedure for pectus excavatum: evolution of techniques and early results on 322 patients. Ann Thorac Surg.

[B20] Croitoru DP, Kelly RE, Goretsky MJ, Lawson ML, Swoveland B, Nuss D (2002). Experience and modification update for the minimally invasive Nuss technique for pectus excavatum repair in 303 patients. J Pediatr Surg.

[B21] Hebra A, Swoveland B, Egbert M, Tagge EP, Georgeson K, Othersen HB (2000). Outcome analysis of minimally invasive repair of pectus excavatum: review of 251 cases. J Pediatr Surg.

[B22] Dzielicki J, Korlacki W, Janicka I, Dzielicka E (2006). Difficulties and limitations in minimally invasive repair of pectus excavatum-6 years experiences with Nuss technique. Eur J Cardiothorac Surg.

[B23] Kim DH, Hwang JJ, Lee MK, Lee DY, Paik HC (2005). Analysis of the Nuss Procedure for Pectus Excavatum in Different Age Groups. Ann Thorac Surg.

[B24] Nuss D (2005). Recent experiences with minimally invasive pectus excavatum repair "Nuss procedure". Jpn J Thorac Cardiovasc Surg.

[B25] Peden CJ, Prys-Roberts C (1991). Capnothorax: Implications for the anaesthetist. Anaesthesia.

[B26] Schalamon J, Pokall S, Windhaber J, Hoellwarth M (2006). Minimally invasive correction of pectus excavatum in adult patients. J Thorac Cardiovasc Surg.

